# Interfacial Effects Between Dental Impression and Die Materials and Their Role in the Internal Fit of Indirect Resin-Based Composite Restorations

**DOI:** 10.3390/dj13040155

**Published:** 2025-03-31

**Authors:** Murillo Weissheimer, João Carlos S. N. Foly, Fabíola G. Carvalho, Eliseu A. Münchow

**Affiliations:** 1 Graduate Program in Dentistry, School of Dentistry, Federal University of Rio Grande do Sul, Porto Alegre 90035-004, Brazil; weiss.muri@gmail.com (M.W.); joaosilva2111@icloud.com (J.C.S.N.F.); 2 Graduate Program in Dentistry, School of Dentistry, Federal University of Juiz de Fora, Governador Valadares 35010-180, Brazil; fabiola.carlo@ufu.br; 3 Department of Conservative Dentistry, School of Dentistry, Federal University of Rio Grande do Sul, Porto Alegre 90035-004, Brazil

**Keywords:** dental internal adaptation, dental impression materials, wettability, surface properties, permanent dental restoration

## Abstract

**Background/Objectives:** This study tested a method for evaluating the internal fit of indirect resin-based composite (RBC) restorations, as well as the influence of different combinations of impression and die materials on the reproducibility of the topography of teeth prepared for indirect RBC restoration. **Methods:** Bovine incisors received flattened and cavitated areas at the cervical and middle thirds of the buccal surface, respectively. The samples were randomly assigned to two groups according to the material used for impression taking (n = 5): irreversible hydrocolloid and polyvinyl siloxane (PVS). Die replicas were obtained with Type IV gypsum or elastomeric material. RBC restorations were fabricated through an indirect technique (test) and a direct-indirect technique as the control. The internal fit of restorations was assessed by measuring the cementation line thickness with a digital caliper (simulated cementation protocol with ultra-light PVS) and validated using scanning electron microscopy (SEM). Surface topography (Sa, Sq, and Sz) was analyzed via optical profilometry, and wettability was assessed through the water contact angle method. The data were analyzed using *t*-test, ANOVA, and Pearson correlation tests (α = 5%). **Results:** The simulated cementation resulted in internal gap values positively correlated to the values from SEM (R^2^ = 0.958; *p* = 0.0102). The internal gap of restorations was not significantly correlated with the discrepancies between the topography of the die and tooth substrate (*p* ≥ 0.067). The combination of irreversible hydrocolloid and gypsum resulted in restorations with the lowest cementation line thickness, although in terms of roughness, this combination was the only one that resulted in significant differences from the control (*p* ≤ 0.028). The internal mean gap values of restorations were significantly correlated to the cumulative wettability difference of materials used during impression taking, fabrication of die replica, and restoration build-up (R^2^ = 0.981; *p* = 0.003). **Conclusions:** The reproducibility of topographical characteristics of the tooth in the die replica did not affect the internal adaptation of indirect RBC restorations, whereas surface wettability of materials presented a more relevant effect on the overall gap formation. The simulated cementation technique tested in the study shows potential as a simpler, cost-effective, and non-destructive method for evaluating the adaptation of indirect RBC restorations.

## 1. Introduction

Resin-based composites (RBCs) are the material of choice for dental restoration in both anterior and posterior teeth, showing adequate physical and mechanical properties [1,2]. The indirect technique using RBCs—when the restoration is fabricated in a cast or a die replica of the tooth cavity—has several advantages over the direct technique, including superior marginal adaptation, reduced polymerization stress, and additional extraoral light-curing [3,4]. According to some studies, the success rate of indirect RBC restorations is superior to 80% at the mid-long term [5,6], without significant differences compared with the direct technique [7]. Nevertheless, the traditional protocol for indirect restoration using RBC is technique-sensitive due to the sequential clinical steps starting with tooth cavity preparation, impression taking, die fabrication, restoration build-up, and then the cementation in the tooth. Notably, any error that occurs during each of the foregoing steps may jeopardize the clinical performance of the restoration.

Even though modern dentistry has benefited from the use of digital scanning for tooth impression and the use of 3D printed resins for die fabrication, the foregoing methods are still expensive and directly dependent on the quality of equipment employed [8,9]. Conversely, the analogical workflow using conventional impression materials (e.g., irreversible hydrocolloid or polyvinyl siloxane/PVS) and die materials (e.g., Type IV gypsum or elastomeric polymers) is more affordable for most dental professionals [10] who are more familiar with the characteristics of these clinical steps. However, the physical compatibility between impression and die materials must be considered, as they have different chemical and wettability characteristics [11]. The study by Patel et al. [12] demonstrated that detail reproduction varied depending on the type of PVS and gypsum, with some combinations resulting in deterioration of gypsum surfaces, suggesting an incompatibility between the two phases if put in direct contact. Also, the flowability and rheological properties of the die can interact differently with the surface of the impression material, influencing the dimension accuracy of the die and the adaptation of the final restoration [13].

Few studies [11,14] have investigated the physical compatibility of impression and die materials through the effects of wettability, suggesting that this factor plays a major role in the final quality of the die replica. According to de Abreu et al. [13], different marginal gap values should be expected even upon the use of materials with similar characteristics. One additional aspect to be considered is the wetting ability of the impression material to the tooth, which may also impact the precision of the impression [15]. In this case, hydrophilicity is recognized as a major influencing factor, as demonstrated elsewhere [16]. No less important, it has been discussed that the topography of the inner area in indirect restorations may influence the trueness and final fit of restorations [17], as imprecise surface reproducibility may lead to a thicker cement line, compromising the fit of the restoration. However, the study by Lövgren et al. [18] revealed that increased surface roughness may result in better internal and marginal fit in the case of metallic restorations, while another study [19] demonstrated that rough surfaces may result in misfitted restorations. To the best of our knowledge, no study has evaluated the optimal combination of impression and die materials for indirect RBC restorations in terms of internal fitting and the role of wettability and topographical characteristics on this outcome.

The evaluation of the internal adaptation of restorations is usually accomplished through micro-computed tomography (µ-CT) or via scanning electron microscopy (SEM) evaluation. SEM is usually used as the election method [20,21], although it requires the sample to be sectioned, thus being a destructive methodology. In turn, µ-CT is a non-destructive method and positively correlated with SEM, although limitations related to specimen radiolucency and magnification power are also reported [22,23,24]. Despite both methods being valid, the need for specific and expensive equipment makes their use more difficult, thereby motivating the search for simpler methodologies. In dentistry, there are other methods reported for measuring the internal fit of restorations, including stereomicroscopy [25], laser microscopy [26], and the replica technique [27,28]. In turn, the latter technique combines the use of light- and heavy-bodied PVS to obtain replicas of the restoration-tooth interface, with the light-bodied PVS simulating the cement space occupied between tooth and restoration during the cementation step. The latter is a validated technique broadly used in prosthodontics, although some shortcomings may also exist, like the need for sectioning the sample and the influence of the seating pressure during handling of the PVS material.

Hence, the aim of this study was first to test a new method for evaluating the internal fit of indirect restorations, and second to investigate the influence of different combinations of impression and die materials on the reproducibility of the topographical characteristics of teeth prepared for indirect RBC restoration. Three null hypotheses were tested: (1) there is no difference in gap distance comparing the two measuring protocols (simulated method and SEM analysis); (2) surface roughness is not correlated with the final accuracy of restorations; and (3) there is not any difference between the wettability of impression, die, and RBC materials.

## 2. Materials and Methods

### 2.1. Sample Size Calculation and Study Design

**Figure 1:**
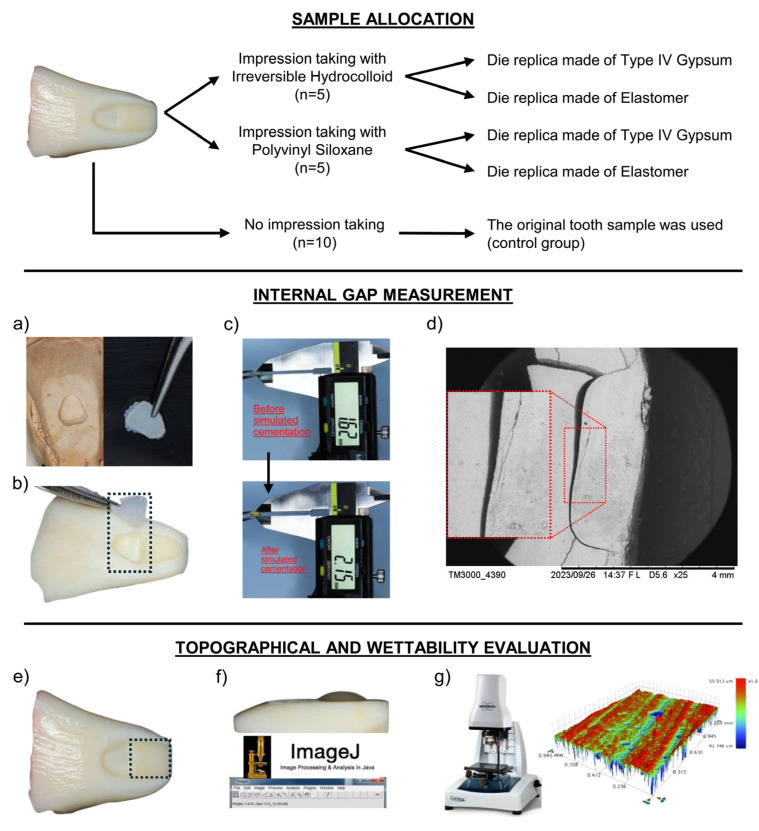
Illustration showing the experimental design tested in the study. Regarding sample allocation, the upper chart shows the two areas prepared in the tooth samples, one at the most cervical area (flat dentin) and the other at the middle third (tooth cavity). The samples were then allocated (n = 5) according to two impression materials (irreversible hydrocolloid or polyvinyl siloxane); the samples in each group were copied twice to obtain two types of die replicas, one made of type IV gypsum and the other made of elastomer. All tooth samples were also tested without impression taking (n = 10), so the restorations were prepared in each tooth cavity (control group) for comparison with the restorations prepared in the die replicas. Concerning the internal gap measurement, resin-based composite (RBC) restorations were fabricated in each tooth and die replica (**a**), verified for adaptation into the tooth cavities (**b**), measured using a digital caliper before and after simulated cementation (**c**), followed by definitive cementation for validation using scanning electron microscopy (SEM) analysis (**d**). The samples (tooth substrate, impression, die, and RBC materials) were also evaluated at the flat dentin area (**e**) using the water contact angle method for wettability analysis (**f**), and through optical profilometry for surface roughness analysis (**g**). In the SEM micrograph, the inset image represents an approximated area showing the cement line measured for validation with the gap values obtained upon the use of the new measuring method tested in the study.

Sample size was calculated based on the internal gap values of a prior study [13], wherein the minimum detectable difference was stipulated to be 100 μm among four independent groups, with an estimated standard deviation of the difference of 45 μm, the test’s power set at 80%, and the type α error as 5%, resulting in a sample size of n = 5 per experimental group. The calculus was performed using the calculator available at https://calculoamostral.bauru.usp.br (accessed on 3 May 2024). Two variable factors with a 2 × 2 factorial distribution were considered, namely the type of impression material (irreversible hydrocolloid and PVS) and the type of die material (type IV gypsum and elastomeric die material). The response variables tested in the study were the internal mean gap of restorations, the roughness of tooth and die samples, and the water contact angle (wettability) of tooth, impression, die, and restorative materials. The experimental design of the study can be observed in Figure 1, which illustrates the allocation of samples and measurements applied.

### 2.2. Sample Preparation and Group Allocation

Bovine incisors were selected, cleaned of debris, and disinfected in 0.5% chloramine-T (Merck, Sao Paulo, Brazil) solution for seven days. The root portion was removed with a low-speed diamond saw (Isomet 1000; Buheler, Lake Bluff, IL, USA), followed by palatal surface grinding with #600-grit silicon carbide (SiC) sandpaper to guarantee an even and parallel position of the tooth. All teeth received two preparations in the buccal surface: first a flat dentin surface was prepared at the most cervical portion of the tooth with a #4138 regular-grit diamond bur (KG Sorensen, Serra, Brazil), followed by polishing with #400- and #600-grit SiC to make the area parallel to the ground; and second a trapezoid-shaped cavity was created at the middle third with a #3131 diamond bur having approximately 5 mm length, 4 mm width, and 1.6 mm depth. To guarantee standardized tooth cavities, the shape of a trapezoid having the foregoing length and width dimensions was marked with a pencil onto the middle third of the buccal surface, and the #3131 bur was identified with a permanent pen onto the ~1.6 mm height mark. All preparations were carried out by one experienced operator and under refrigeration, replacing the bur every five preparations. The samples were cleaned using pumice and then randomly assigned to two groups according to the impression material used to copy the flat portion and the tooth cavity areas: irreversible hydrocolloid (AlgiGel; Maquira) and PVS (Scan Light; Yller Biomaterials). The randomization of samples was performed using a free web sort calculator available at https://www.sorteiogo.com/pt (accessed on 27 May 2024). To that end, each sample was first positioned side by side, receiving a sequential number. The total number of samples was added in the calculator tool, which was run to result in a random sequence of numbers within the experimental groups. Table 1 shows information on the manufacturers, composition, and instructions for use of materials.

In the irreversible hydrocolloid group, two impressions were taken from each sample to obtain two independent die replicas, with the impressions being poured within a maximum of 5 min after impression taking to guarantee the reliability of the copy. The dies were fabricated with Type IV gypsum (Durone IV; Dentsply) or elastomeric material (Scan Die; Yller Biomaterials). In the PVS group, only one impression of each tooth sample was taken, and the impression was poured twice (once with each die material) after 2 h since impression taking, being cleaned and checked for the existence of remnants of the previously poured die material. Before pouring the elastomeric material, the PVS impression was covered with an isolating agent (Isolator; Yller Biomaterials, Pelotas, Brazil) as per the instructions of the manufacturer. The impressions were made using a partial metal tray, which was applied perpendicularly to the center-positioned tooth samples; the die materials were handled according to the manufacturers’ directions (Table 1). After the setting time of each material, the two phases (impression and die) were separated carefully.

### 2.3. Fabrication of Restorations

RBC restorations were fabricated from the tooth samples using the direct-indirect technique proposed by Fahl Jr [29], and the corresponding dies were used to fabricate the restorations following the indirect technique. The direct-indirect technique was simulated in the cavities of all dental samples, taking these restorations as the control group since no impression taking or die fabrication was necessary, serving as the exact adaptation of the RBC within the original tooth cavities for comparison. For the control restorations group (n = 10), the tooth cavities were first isolated with a thin layer of water-soluble gel (K-Y, Gel, Semina; Sao Paulo, Brazil) and then filled with a single increment of bulk-fill RBC (Opus Bulk Fill; FGM), which was adapted to the cavity using a titanium Thompson spatula (Indusbello; Londrina, Brazil). Photoactivation was performed for 40 s with a light-emitting diode (LED) curing unit (Raddi Cal, SDI; Bayswatter, Australia). The restoration was removed from the cavity, and the marginal excess was finished with a #15 scalpel blade. In turn, the cavities replicated in the dies were filled with uncured RBC, simulating the indirect technique. A thin layer of water-soluble gel was applied to the cavities of the gypsum dies, whereas the cavities from the elastomeric dies did not require isolation due to the polymeric nature of the material. After photoactivation, the restorations were separated from the cavities, and the marginal excess was removed (finished) with a #15 scalpel blade.

### 2.4. Evaluation of the Internal Adaptation of Restorations

To assess the difference in fitting and internal adaptation of restorations, the following method was applied. First, the thickness of each restoration was measured in two areas at the axial wall using a 150 mm digital caliper (Model 500-196-30 Absolute, Mitutoyo; Jundiaí, Brazil) with an accuracy of 0.01 mm; one measurement was taken at the most cervical region of the axial wall, and the other at the most incisal region of restorations. The thickness values were tabulated in an Excel spreadsheet (Microsoft Office 2016).

The restorations fabricated in the tooth samples (direct-indirect control group) and those in the dies for each experimental group were individually cemented to the tooth cavities using a simulated cementation method with ultralight PVS (Futura; DFL) as the ‘cementing material’ [27]. The PVS was manipulated according to the manufacturer’s instructions and applied into the cavities using an automatic mixing tip. This process was repeated three times for each tooth cavity, one for each subgroup: (1) restorations obtained via the direct-indirect technique (control); (2) restorations obtained via the indirect technique fabricated in gypsum dies; and (3) restorations obtained via the indirect technique fabricated in elastomeric dies. After polymerization of the ‘cementing material’, any marginal excess was removed from the buccal surface with a #15 scalpel blade.

The restorations from each corresponding subgroup were removed from the cavities and measured for the thickness of the cementation line at both cervical and incisal areas, as described earlier. The measurement included both the thickness of the restoration itself and the ‘cement layer’. An average value was taken from the cervical and incisal values of each restoration. The difference between the final thickness (restoration + ‘cement layer’) and the baseline thickness (before the simulated cementation) of each restoration was calculated and expressed in μm, representing the mean internal gap (’cementation line’) for each group. A lower value indicates better fitting and internal adaptation of the restoration to the tooth cavity, whereas a higher value indicates greater maladjustment (gap) of the restoration. In this study, an internal gap length of up to 120 µm was considered clinically acceptable, as suggested elsewhere [13].

### 2.5. Scanning Electron Microscopy (SEM) Analysis

To validate the new cementation method tested in this study, samples from each group were evaluated with SEM. The restorations were permanently cemented to the respective original teeth using a flowable self-adhesive resin composite (Yflow SA; Yller Biomaterials). After photo-activation with the LED for 40 s, the restored teeth were sectioned using a precision cutting machine (Isomet 1000) along the longitudinal axis to obtain thin slices (~1.5 mm thick). The slices were sputter-coated with Au and then analyzed under SEM (TM3000, Hitachi; Chiyoda, Japan). The images were processed using ImageJ (version 1.53 k) to measure the thickness of the cementation line, which was correlated to the ‘cementation line’ of each group of the study.

### 2.6. Optical Profilometry Analysis

The flat area of dentin was evaluated in all tooth samples and dies using an optical profilometer (model GTK M; ContourGTK, Bruker, Atibaia, Brazil). Three measurements were taken from each sample, obtaining the values for the Sa (arithmetic mean of surface roughness), Sq (mean quadratic value of the defined area, being equivalent to the standard deviation of heights) and Sz (the sum between the highest peak and the deepest valley in the defined area) parameters. Tridimensional (3D) images were also collected and qualitatively analyzed.

### 2.7. Surface Wettability Analysis

The surface wettability of each dental sample, impression materials, die replicas, and RBC was evaluated using the water contact angle (θ) method, as described elsewhere [30]. Briefly, a drop of distilled water (~5 μL) was dispensed on the central portion of the cervical flattened area of each substrate; for the measurement of impression and RBC materials, flat samples were prepared by letting the material cure in-between two glass slides (chemical curing for the impression materials, and light-curing for the RBC). A photographic image was taken within 5 s with a professional camera (EOS Rebel T3i; Canon, Huntington, NY, USA) coupled with an EF-S 105 mm lens (Canon) and circular flash (Canon). The image collection distance was standardized for all tested substrates at 20 cm. The images (n = 5) were processed using ImageJ (National Institute of Health; Bethesda, MD, USA) to measure the θ obtained between the water drop and the surface of the sample. Two measurements were taken per image/sample: one on the left side of the drop and one on the right. The mean θ was calculated and expressed in degrees (°); values below 90° mean a hydrophilic material, while values above 90° indicate a hydrophobic material.

### 2.8. Statistical Analysis

The 3D images obtained from the optical profilometry analysis were qualitatively evaluated, while the other quantitative data were evaluated using SigmaPlot version 12.0 (Systat). The data were all normally distributed, so they were analyzed using *t*-tests (to compare the topographical measurements between experimental control groups), and two-way repeated measures analysis of Variance (ANOVA) and Tukey’s post hoc test were used to compare the effects of the variable factors tested in the study. Correlation tests were performed on all the collected data. Pearson’s correlation test was conducted between the internal gap values from the simulated cementation method and the respective values obtained via SEM evaluation (validation test). Also, gap values were correlated to the discrepancies between the topographical characteristics of the die and dental substrates. Finally, the data from the simulated cementation method were correlated with the cumulative wetting difference between materials used during impression taking, die fabrication, and RBC restoration. In this case, the cumulative wetting difference (Δθ) was calculated separately for the restorations obtained via the direct-indirect or indirect techniques, using the following equations:

Δθ_direct-indirect technique_ = θ_RBC_ − θ_tooth_ (1)

Δθ_indirect technique_ = ((θ_impression_ − θ_tooth_) + (θ_die_ − θ_impression_) + (θ_RBC_ − θ_die_)). (2)

The level of significance was set at α = 5% for all the analyses.

## 3. Results

### 3.1. Validation of the Simulated Cementation Method with SEM

**Figure 2:**
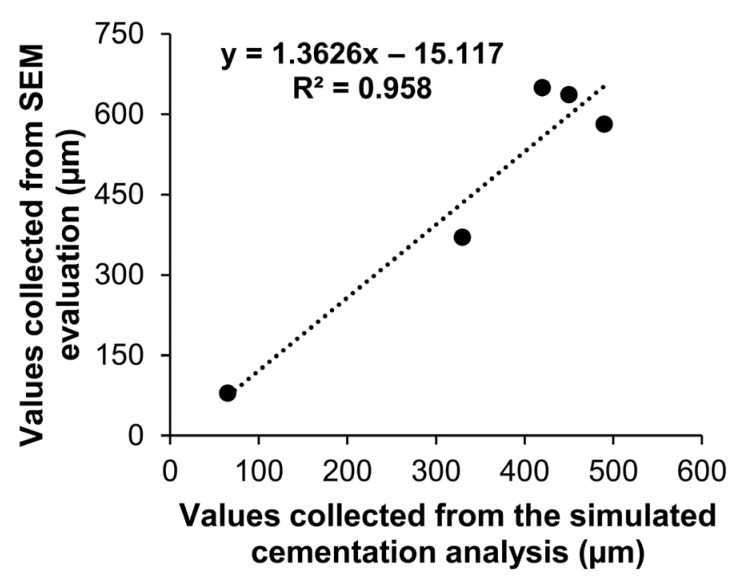
Graph showing the correlation between the gap values collected from SEM evaluation and those collected from the simulated cementation method used in the study.

The mean gap values obtained for the experimental groups of this study using the simulated cementation method with ultralight PVS were positively correlated to the mean gap values calculated through SEM evaluation, as demonstrated in Figure 2. The correlation test revealed an excellent relationship between the values (R^2^ = 0.958), showing a statistically significant correlation (*p* = 0.0102), validating the simulated cementation method of the study.

### 3.2. Internal Fit of Restorations

The results for the internal adaptation of restorations (mean gap values) of all groups are shown in Table 2. The variable factors were not significant (*p* > 0.05), although their interaction was statistically significant (*p* = 0.006). The gypsum die obtained from the PVS impression showed higher gap values than the counterpart obtained from the irreversible hydrocolloid impression (*p* = 0.024); whereas the elastomeric die obtained from the irreversible hydrocolloid impression was the one with the highest internal gap values of the study (496.7 ± 23.6 µm) and higher than that from the elastomeric die obtained with PVS impression (*p* = 0.043). Among the dies obtained with irreversible hydrocolloid impression, the elastomeric die had a significantly thicker cementation line compared to the gypsum die (*p* < 0.001); the dies obtained from PVS impression showed similar internal gap values (*p* = 0.868). Compared to the control, the dies resulted in similar gap values, except for the elastomeric die from the irreversible hydrocolloid group, which had a greater cementation line than the control (*p* = 0.002).

### 3.3. Surface Reproducibility

The Sa values of prepared teeth at baseline ranged from 1.98 µm to 3.98 µm, with no significant differences between samples from the irreversible hydrocolloid and the PVS groups (*p* ≥ 0.06). Table 3 shows the results of the topographical measurements obtained from the optical profilometry for all groups tested in the study, with the ‘type of impression material’ and the ‘type of die material’ as variable factors. Compared to the control group (tooth samples), the different dies resulted in similar Sa, Sq and Sz values, except for the gypsum die obtained from the impression with irreversible hydrocolloid, which showed higher Sa (4.23 ± 0.73 µm; *p* = 0.028), Sq (8.02 ± 2.50 µm; *p* = 0.024), and Sz (120.5 ± 34.5 µm; *p* = 0.018) values than the control (Sa = 2.80 ± 0.73 µm; Sq = 3.85 ± 1.15 µm; and Sz = 69.7 ± 16.8 µm).

Regarding the Sa parameter, the type of die was a significant factor (*p* = 0.017), with no statistical interaction between the factors (*p* = 0.627). The elastomeric die from the PVS group presented lower Sa values (2.19 ± 0.03 µm) than the counterpart made of gypsum (3.67 ± 0.73 µm) (*p* = 0.036). For the Sq parameter, both variable factors were significant (*p* ≤ 0.033), although their interaction was not significant (*p* = 0.969). The Sq values were significantly lower in the elastomeric dies, regardless of the impression material (*p* ≤ 0.043). Concerning the Sz parameter, the variable factors were not significant (*p* ≥ 0.092), and they did not interact statistically with each other (*p* = 0.707). No differences were found among the tested groups in Sz values.

**Figure 3:**
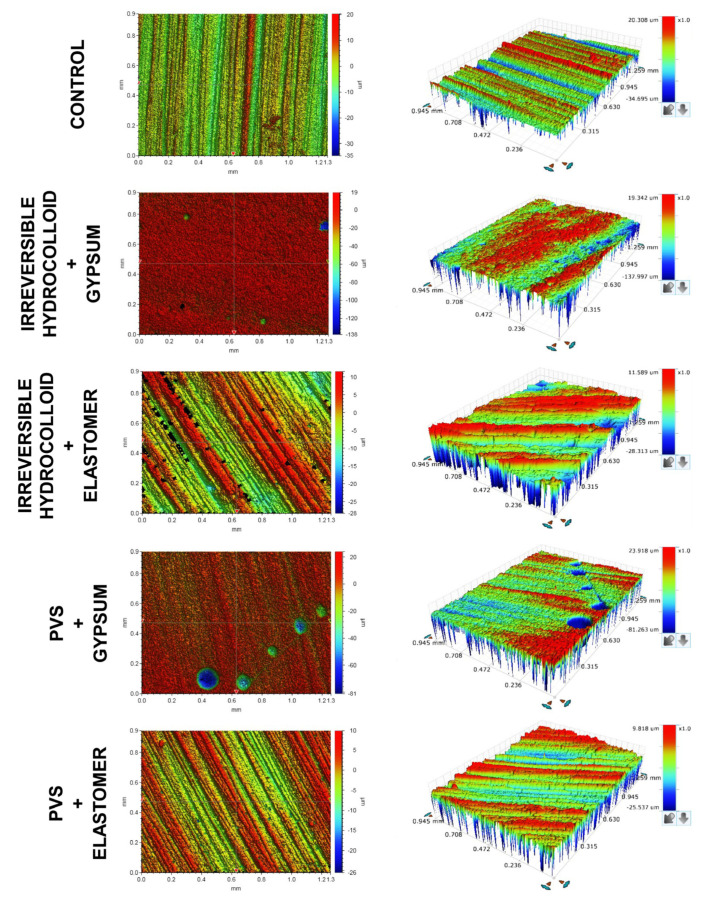
2D and 3D images from the optical profilometry analysis conducted for all groups tested in the study. PVS: polyvinylsiloxane.

Figure 3 presents the 3D images of the topography of the groups tested in the study. The dentin substrates showed a uniform topography, with peaks and valleys ranging from 20.3 µm to −34.7 µm. The gypsum samples demonstrated similar peaks to the tooth control (~19 µm) but considerably deeper valleys (varying from −81.3 µm to −138 µm); also, the occurrence of a porous surface with round-shaped defects in the gypsum dies was observed, from both irreversible hydrocolloid and PVS groups. Regarding Sz, despite the notably higher value for the gypsum group, it was not statistically significant. Qualitative analysis of the 3D images revealed higher peaks and deeper valleys for the gypsum dies.

The combination of irreversible hydrocolloid and gypsum was the only one that resulted in statistically significant differences regarding Sa, Sq, and Sz parameters compared to the control group. Qualitative assessment of Figure 3 demonstrates that by combining these two materials, the surface pattern is different from the tooth sample. The internal mean gap values of restorations were not significantly correlated with the discrepancies between the topography of die and tooth substrate: Sa parameter (R^2^ = −0.345, *p* = 0.328); Sq parameter (R^2^ = −0.552, *p* = 0.098); and Sz parameter (R^2^ = −0.600, *p* = 0.067).

### 3.4. Wettability Properties

**Figure 4:**
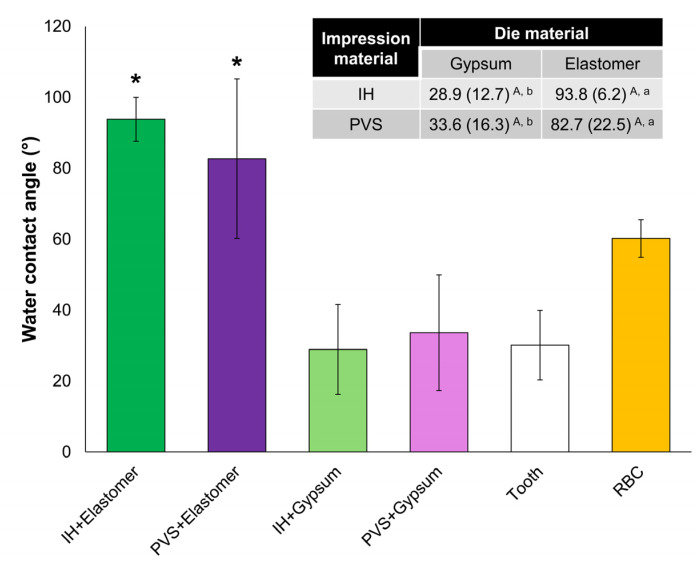
Graph in bars showing the water contact angle (wettability property) of the die replicas obtained in gypsum and elastomeric material after impression taking with irreversible hydrocolloid (IH) or polyvinylsiloxane (PVS). The graph also shows the wettability of the tooth substrate (control) and of the resin-based composite (RBC) tested in the study. The in-set table shows the mean and standard deviation values for the water contact angle of groups and the results of the statistical analysis (two-way ANOVA; Tukey’s post hoc, *p* < 0.05), with different uppercase letters in the same column and lowercase letters in the same row indicating statistically significant differences between the impression materials or between the die materials, respectively. The presence of an asterisk above the bars indicates a statistically significant difference between the respective group and the tooth control (*t*-test, *p* < 0.05).

The results for the wettability properties of all groups are shown in Figure 4. The type of die material was the only significant factor (*p* ≤ 0.001), with no significant interaction between the factors (*p* = 0.405). The elastomeric dies demonstrated higher θ values than the gypsum dies (*p* ≤ 0.005), regardless of the type of impression material. The θ values for the irreversible hydrocolloid and PVS impressions were 23.7° ± 5.4° and 95.7° ± 11.5°, respectively, with significant differences (*p* < 0.001). The tooth sample presented a θ = 30.1° ± 9.8°, and the RBC tested in the study showed a θ = 60.2° ± 5.3°, which were statistically lower than the elastomeric dies (*p* ≤ 0.001).

**Figure 5:**
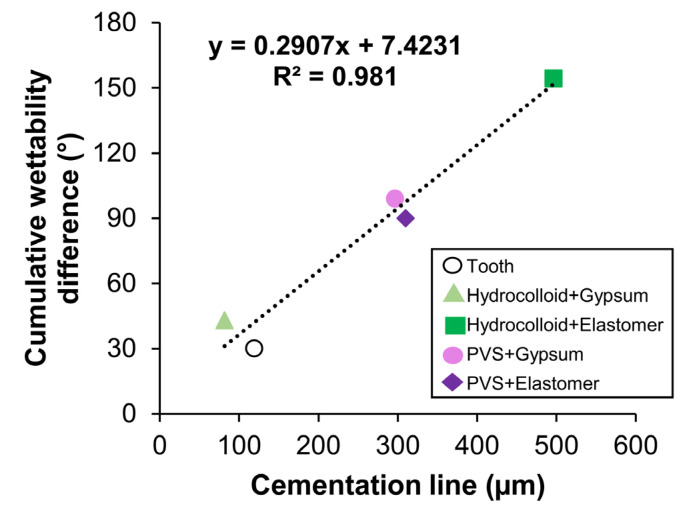
Graph showing the correlation between the cumulative wettability difference and the cementation line of restorations. PVS: polyvinylsiloxane.

The internal mean gap values of restorations were significantly correlated with the cumulative wettability difference (Δθ) resulting from the combination of different impression, die, and restorative materials, showing a positive relationship (R^2^ = 0.981; *p* = 0.003) as presented in Figure 5.

## 4. Discussion

This study investigated a method for evaluating the internal fit of RBC restorations that would not require the sectioning of restored samples. Of note, the mean gap values measured with the present simulated cementation protocol showed an excellent relationship (almost perfect correlation as observed in Figure 2) with the gap values assessed through SEM evaluation, so the first null hypothesis of the study was accepted.

To the best of our knowledge, this is the first study that proposed this alternative and non-destructive way of measuring the adaptation of RBC restorations. Even though SEM and µCT analyses are considered the gold standard methods, they require expensive equipment that could hamper the assessment of internal/marginal fit of restorations worldwide, so we targeted a simpler, cost-effective, and non-destructive method. The simulated cementation protocol consisted of using a material that filled the gap between the substrate and RBC restoration, while also being easy to remove along with the restoration without tearing. Other studies showed the reliability of using light-bodied PVS as a cement material, like in the replica technique [27,31], but limitations were already discussed, such as the possible inaccuracy resulting from finger pressure during the cementation step. The same shortcoming was also expected to occur in our method, but all procedures were performed by only one experienced dentist, so the cementation protocol, handling of restorations, and thickness measurements were standardized by the same operator, minimizing potential bias.

One may also suggest that the simulated cementation method is not reliable since it may substantially reduce the number of evaluation points, and that it may depend on the stabilization of the ‘cement material’ (i.e., the light-bodied PVS) to the inner face of the RBC restoration. First, it is noteworthy that even under SEM evaluation, gap measurement does not represent the true circumferential fit of the restoration, since slices from the original sample are cut to be microscopically analyzed; the only method that allows full gap measurement is µCT [22,24] due to the creation of 3D images of the restorative interface, although limitations related to specimen radiolucency and magnification power may also be relevant to consider [22,23,24]. Second, the PVS used in our method as ‘cement material’ could not be stabilized with heavy-bodied PVS, like in the replica technique [27], as it would hamper the evaluation of the thickness for the restoration + cement composition. Again, the restorations were handled as gently as possible and by the same operator to allow the adequate measurement of the cement line plus the original thickness of restorations.

The second null hypothesis of the study was that surface roughness is not correlated with the final accuracy of restorations. Overall, the die replicas made of gypsum showed the roughest condition, especially the dies that originated from an impression with irreversible hydrocolloid (Table 3), which was the only combination that differed statistically from the topographical pattern of the tooth. However, the latter combination had the best internal fit of the study (Table 2), so roughness was not correlated to restoration accuracy, accepting the second null hypothesis. The idea that imprecise surface reproducibility may lead to a thicker cement line and consequently to a misfitted restoration is not new in dentistry [17,19]. Nonetheless, considering that an up to 120 µm thick cement line is clinically acceptable [32,33], surface roughness consisting of peaks and valleys within that threshold would not be problematic. Indeed, only the gypsum dies obtained through the impression with irreversible hydrocolloid had a Sz value exceeding the 120 µm threshold, and despite this rough topography, the internal fit of restorations was not impacted negatively.

It is worth observing in Figure 3 that the scratched surface of the tooth substrate (derived from SiC grounding) was replicated in the surface of the elastomeric dies, suggesting their ability to reproduce topographical characteristics. Conversely, the scratchy surface of the tooth was less pronounced in the gypsum dies obtained through impression with PVS, and there was not any clear sign of scratches in the gypsum replicas obtained via impression with irreversible hydrocolloid, reinforcing the fact that some chemical/physical interaction occurs upon the combination of impression and die materials, as demonstrated in previous studies [11,12,13]. Indeed, gypsum deterioration was already reported when it was poured into molds made of PVS [11,12]. In our study, the combination of gypsum and irreversible hydrocolloid was the one in which the surface presented with round-shaped defects.

One may understand that gypsum has a porous nature [34], absorbing humidity from the mold during chemical setting, so the topography of the die was altered in this experimental group. Even so, it should be considered that digitization of dental materials on optical profilometers can be affected by the color and transparency of the material, with darker materials showing rougher values than lighter counterparts [35]. This was not a concern in our study since both die materials under testing (gypsum and elastomer) had a light appearance, so roughness evaluation was probably not affected by the laser scans. Notably, another study did not demonstrate any interaction between optical scanning and impression/die materials regardless of color and opacity [36], so future studies could investigate further this aspect.

The present findings highlight that the physical incompatibility between materials used during tooth cavity impression, die replica fabrication, and restoration build-up is an important factor to be considered. Wettability is the characteristic of a material that concerns the tendency of a fluid to spread or adhere to its surface. When two solids come into contact with each other, such as the interaction between impression materials and the tooth surface or between die and impression materials, a complex set of factors could affect the foregoing interaction, such as the materials’ characteristic of being hydrophobic or hydrophilic [16]. Hydrophilic and hydrophobic materials tend to repel each other, whereas materials with similar superficial tension tend to be attracted, potentially resulting in a better replication of the inner characteristics of the surface [15,37]. Irreversible hydrocolloids have a surface wettability similiter to the tooth [10], which may lead to better interaction and more adequate physical compatibility. Conversely, PVS and elastomers are more hydrophobic [15], suggesting physical incompatibility with hydrophilic matter like the tooth and hydrocolloids [38]. Likewise, the surface wettability of gypsum is like irreversible hydrocolloids, so they would not repel each other while one is being poured into the other, explaining the good internal fit verified for restorations prepared using both materials.

It is noteworthy that the control group of our study presented mean internal gaps within the 120 µm thick threshold, but also with some important variance. Here, it is of utmost importance to consider that these restorations were prepared by applying the RBC directly to the tooth cavity [29], so the RBC itself would copy the exact shape and internal dimensions of the cavity, perhaps resulting in the best fit for the final restoration. However, this was not the entire case since some gap values exceeded the clinically acceptable threshold, as mentioned before. A possible explanation relies on the fact that before RBC application, the tooth cavities were isolated with a thin layer of water-soluble gel to prevent the accidental retention of the RBC, so the isolator could have occupied some space in some of the samples, causing the occurrence of some maladjustments during cementation. This aspect should be further investigated in the future.

Remarkably, our study is the first to demonstrate that the greater the total difference in wettability between materials used during tooth impression, die fabrication, and restoration build-up, the greater the maladaptation of the indirect RBC restoration (Figure 5). Therefore, the use of materials with similar wettability properties should be considered at each step of the procedure to minimize incompatibility between the material phases, resulting in a better restoration fit. Based on surface wettability alone as a decision factor for choosing impression and die materials, the combination of irreversible hydrocolloid for impression taking and gypsum for die fabrication seems to be the most appropriate combination due to their better physical compatibility. A similar trend was observed comparing the low cumulative wettability difference between the tooth and the RBC in the direct-indirect technique (control), which resulted in reduced gap values. Given this finding, the third null hypothesis of the study, that there is no difference between the wettability of impression, die, and RBC materials, was rejected.

In our study, most restorations did not fit perfectly into the tooth cavities. When a restoration made using the indirect technique is thinner than the original cavity, the cement material will fill this space, compensating for the reduced thickness of the restoration. The American Dental Association defines the ideal cementation line between restoration and tooth should be of a maximum of 25 µm (using zinc phosphate cement), with a cementation line up to 120 µm considered acceptable for other cementing agents [32,33]. In this study, cementation line thickness was defined as the difference between the thickness of the cemented and pre-cemented restoration. Notably, only restorations made in gypsum, obtained via irreversible hydrocolloid impression, had an average cementation line lower than 120 µm, supporting the hypothesis of physical interaction between the materials. This emphasizes the importance of choosing compatible impression and die materials during the fabrication of indirect RBC restorations. The highest maladaptation values were observed when combining irreversible hydrocolloid and elastomeric die materials, reinforcing the effect of their physical incompatibility. Similarly, the combination of PVS and gypsum die also resulted in an unsatisfactory average cementation line thickness. Convergently, previous studies point out that the cementation line should be as thin as possible, reducing the chance of marginal infiltration of indirect restorations and their consequent clinical failure [39].

The present study has several limitations. During the simulated cementation process using ultra-light PVS, adaptation levels were measured with a digital caliper, so variations in pressure during measurement could lead to deviations from the true value. The wettability of the ultra-light PVS used as ‘cement material’ should also be considered as an influencing factor, as it may cause physical incompatibility with the tooth and the RBC restoration, although applying digital pressure during the simulated cementation (as in the clinical scenario) could have minimized any repellency between materials. In this respect, alternative materials could be investigated in place of PVS for cementation purposes. No less important, one may have the concern that bovine incisors were tested in this study, so the results would not relate to human teeth; however, bovine teeth have similar physical properties to human teeth [40], and since our main goal was to have a viable substrate to prepare and apply impression taking materials, the choice for using this source of dental tissue should not have influenced the results, keeping it a reliable source for measuring the internal gap of RBC restorations.

The RBC used in our study is a class of material from a direct technique. While a commercial indirect RBC should have been more interestingly used in this study, direct RBCs have also been applied in indirect techniques [41]. Considering that our control group was based on the direct-indirect technique proposed by Fahl et al. [29], we chose to use a direct RBC to fabricate the indirect restorations. The wide range of dental products available in the market, with notable differences in composition between brands, also underscores the need for further studies using different materials. Last, the present study did not consider the effects of disinfection solutions on the molds obtained during impression taking; clinically speaking, irreversible hydrocolloids and PVS materials may change dimensional stability upon disinfection process [42], so the interaction with die materials could also be impacted, influencing the overall accuracy of the final RBC restoration. Future studies are warranted to assess the cement line thickness of restorations prepared under more similar clinical conditions to those simulated here.

Despite several possible limitations, the present study has one strength regarding the use of the same tooth sample for the cementation of three sets of restorations, i.e., the direct-indirect control group and the restorations fabricated in the gypsum and elastomer die replicas. This guaranteed some dependency among the data, thus requiring a lower number of samples per group, as informed during sample size calculation. Also, the possibility of using the same tooth cavity to cement the restorations of three distinct groups minimized bias due to tooth preparation. According to Haddadi et al. [27], even small differences among teeth prepared for indirect restoration may affect final fitting, so the use of the same tooth for more than one cementation (only possible due to the simulated cementation method tested here) seemed beneficial. Usually, other methods available for measuring the internal gap of restorations use independent samples for each group, so the results are always dependable on the type and quality of the sample; in our study, we could focus on the effects of the combination of different materials in the internal fit of indirect and direct-indirect RBC restorations.

## 5. Conclusions

This study highlights the critical role of physical compatibility between impression, die, and restorative materials in achieving optimal internal adaptation of indirect RBC restorations. While surface topography replication in dies did not significantly affect the adaptation of indirect RBC restorations, the cumulative wettability difference among materials used in the process was strongly correlated with the adaptation of restorations.

The restorations prepared in gypsum dies obtained through irreversible hydrocolloid impression had the lowest cementation gap, opposed by the restoration build-up in elastomer dies obtained through irreversible hydrocolloid, which had the highest internal gap of the study.

The simulated cementation protocol proposed in this study can be an alternative non-destructive method of measuring the adaptation of indirect RBC restorations, although some limitations should be considered depending on the materials under evaluation.

## Figures and Tables

**Table 1:** Composition and application mode of the materials tested in the study.

Material	Category (Application in Study)	Manufacturer	Composition	Application Technique
Algi-Gel Type II	Irreversible hydrocolloid (impression taking)	Maquira (Maringá, PR, Brazil)	Diatomite, potassium alginate, calcium sulfate, magnesium oxide, sodium pyrophosphate, potassium fluortitanate, polyethylene glycol, and flavoring	Vigorous spatulation of the powder-liquid ratio in rubber mixing bowl; after homogenization, the material was inserted into a tray and positioned on the tooth sample until setting as suggested by the manufacturer.
Scan Light	Polyvinyl siloxane (impression taking)	Yller Biomaterials (Pelotas, RS, Brazil)	Base: polyvinylsiloxane, polydimethyl-methylhydrogen siloxane, hydrocarbons, silica, and pigments Catalyst paste: vinyl polysiloxane, hydrocarbons, silica, and platinum complex	A dispensing gun was used to incorporate the material, inserting the material onto the tray with the help of a mixing tip. Working and setting times followed in accordance with the product manual.
Durone IV	Type IV gypsum (die fabrication)	Dentsply Sirona (São Paulo, SP, Brazil)	Calcium sulfate, alpha hemihydrate, and dye	The powder-liquid ratio was applied to a plaster vibrator (under vacuum) and applied to the mold until the entire surface was filled, obtaining a thickness similar to that of a tooth.
Scan Die	Polyvinyl siloxane (die fabrication)	Yller Biomaterials (Pelotas, RS, Brazil)	Base: polyvinylsiloxane, polydimethylmethylhydrogen siloxane, hydrocarbons, silica, and pigments Catalyst paste: vinyl polysiloxane, hydrocarbons, silica, and platinum complex	Applied with a dispensing gun, inserting it over the molds until the entire surface is filled, obtaining a thickness like that of tooth. When used on the polyvinyl siloxane mold, an insulator was applied, which was sprayed onto the mold and allowed to dry.
Opus Bulk Fill	Resin-based composite (fabrication of restorations)	FGM (Joinville, SC, Brazil)	Urethan dimethacrylic monomers, coinitiator, photoinitiator, stabilizers	One increment of up to 2 mm thick was added to the tooth samples and cavities replicated in the dies, followed by light activation for 40 s.
Futura AD	Polyvinyl siloxane (cement material used in the simulation technique)	DFL (Rio de Janeiro, RJ, Brazil)	Polymethyl siloxane, and sílica	A dispensing gun was used to incorporate the material and to apply it into the tooth cavity with the help of a mixing tip. The restoration was then applied to the cavity, and pressure was applied using a metallic spatula. Working and setting times followed the instructions of the manufacturer.
Yflow SA	Self-adhesive resin-based composite (cement material used for permanent cementation of restorations)	Yller Biomaterials (Pelotas, RS, Brazil)	Inorganic fillers, acid monomers (methacryloyloxydecyl-di-hidrogen phosphate and glycerol phosphate dimethacrylate), methacrylate monomers, pigments, initiators, and stabilizers	The material was applied into the tooth cavity using the automatic tip of the product, followed by positioning of the restoration and light activation for 40 s.

**Table 2:** Mean and standard deviation (SD) values for the fitting characteristics obtained with each group tested in the study, varying in terms of impression and die materials.

Parameter	Impression Material	Die Material		Control (Tooth)
		Gypsum	Elastomeric	
Internal gap (µm)	Irreversible Hydrocolloid	81.7 (42.5) ^B, b^	**496.7 (23.6) ^A, a^**	119.2 (132.9)
	PVS	296.7 (71.1) ^A, a^	310.0 (168.9) ^B, a^	

The values given in **bold** indicate that there is a statistically significant difference between the respective group and the control group of the study (*t*-test; *p* < 0.05). Distinct uppercase letters in the same column indicate statistically different values between impression materials, whereas distinct lowercase letters in the same row represent statistically different values between the die model materials (two-way Repeated Measures ANOVA and Tukey’s post hoc test; *p* < 0.05).

**Table 3:** Mean and standard deviation (SD) values for the surface characteristics tested in all groups of the study, varying in terms of impression and die model materials.

Parameter	Impression Material	Die Material		Control (Tooth)
		Gypsum	Elastomeric	
Sa (µm)	Irreversible Hydrocolloid	**4.23 (0.73) ^A, a^**	3.15 (0.21) ^A, a^	2.80 (0.73)
	PVS	3.67 (0.73) ^A, a^	2.19 (0.03) ^A, b^	
Sq (µm)	Irreversible Hydrocolloid	**8.02 (2.50) ^A, a^**	4.40 (0.89) ^A, b^	3.85 (1.15)
	PVS	5.15 (1.46) ^A, a^	2.78 (0.11) ^A, b^	
Sz (µm)	Irreversible Hydrocolloid	**120.5 (34.5) ^A, a^**	72.3 (47.9) ^A, a^	69.7 (16.8)
	PVS	75.5 (13.7) ^A, a^	42.1 (1.6) ^A, a^	

The values given in **bold** indicate that there is a statistically significant difference between the respective group and the control of the study (*t*-test; *p* < 0.05). Distinct uppercase letters in the same column indicate statistically different values between impression materials, whereas distinct lowercase letters in the same row represent statistically different values between the die model materials (two-way Repeated Measures ANOVA and Tukey as post hoc test; *p* < 0.05).

## Data Availability

All the data presented in this study are available within the article.
